# Skin and tissue changes after spinal cord injury-A scoping review

**DOI:** 10.1038/s41394-026-00746-0

**Published:** 2026-08-15

**Authors:** Leander G. Melin, Knaerke Soegaard, Fin Biering-Soerensen, Dimitri Beeckman, Jens A. Soerensen

**Affiliations:** 1 Research Unit for Plastic Surgery, Odense University Hospital, Odense, Denmark; 2 Department of Clinical Research, University of Southern Denmark, Odense, Denmark; 3 Department of Clinical Medicine, University of Copenhagen, Copenhagen, Denmark; 4 Department for Brain and Spinal Cord Injuries, Bodil Eskesen Centre, Rigshospitalet, Copenhagen, Denmark; 5 Skin Integrity Research Group (SKINT), University Centre for Nursing and Midwifery, Department of Public Health and Primary Care, Faculty of Medicine and Health Sciences, Ghent University, Ghent, Belgium; 6 Swedish Centre for Skin and Wound Research (SCENTR), School of Health Sciences, Faculty of Medicine and Health, Örebro University, Örebro, Sweden

**Keywords:** Skin manifestations, Spinal cord diseases

## Abstract

**Study design:**

Scoping review.

**Objectives:**

Spinal cord injury causes complex and severe changes in affected skin and underlying tissues, increasing the risk of pressure ulcer development and impeding wound healing. This scoping review provides an updated overview on skin and tissue changes after spinal cord injury. Given the complexity and inconsistent evidence of the topic, this review outlines current knowledge and highlights research gaps.

**Methods:**

A comprehensive literature search was conducted in Medline, Embase, Cochrane Library, and SCOPUS databases. Studies written in English addressing skin and tissue changes after spinal cord injury were included, with no restrictions on study type or publication year. Two reviewers independently screened the studies, and eligible articles were organized into the following six subgroups: 1) histopathology, 2) ultrasonography, 3) biomechanical properties, 4) skin perfusion, 5) sebum- and sweat gland excretion, and 6) microbiota.

**Results:**

Out of 5387 articles screened, twenty-three met the inclusion criteria. Findings revealed epidermal atrophy characterized by progressive thinning over time, while the dermal layer demonstrated fibrosis and reduced collagen synthesis. Results on skin thickness were inconsistent, though increased tissue stiffness was consistently reported. Skin perfusion was compromised, with diminished reactive hyperemia, impaired vascular autoregulation, and reduced spontaneous skin oxygenation.

**Conclusion:**

Skin and tissue affected by spinal cord injury exhibit compromised quality with characteristics of aged and injured skin. Consequently, the skin is less able to resist pressure, shear stress, and ischemia. The reviewed literature reveals substantial gaps in knowledge, underscoring the need for further research to improve clinical care and management.

## Introduction

Spinal cord injury (SCI) represents a complex and severe medical condition, often resulting in permanent motor and sensory impairment as well as autonomic dysfunctions [1]. While the primary lesion in the spinal cord is crucial for the immediate symptoms and loss of function, subsequent tissue changes that occur in the affected dermatome warrant further attention. The skin and underlying connective tissues are of particular interest due to their essential role in maintaining structural and functional integrity, which is crucial for pressure ulcer prevention, effective wound healing, and the regulation of physiological skin and tissue homeostasis [2].

Following an SCI, the skin in areas at and below the affected spinal cord segments often exhibit a range of pathological changes due to denervation [3]. These include alterations in metabolic and neural mechanisms regulating cutaneous microcirculation [4], leading to reduced oxygenation and impaired wound healing due to a decreased inflammatory phase response [2].

Additionally, a reduction in fibroblast activity affects the extracellular matrix formation, causing a decrease in collagen synthesis, leaving the skin and connective tissue vulnerable to shear stress and continuous pressure [2]. Thus, physiological skin changes following SCI may not only increase the risk of pressure ulcer development but also impair wound healing once ulcers have formed [2, 5].

Individuals with SCI face an elevated lifetime risk of developing pressure ulcers – a serious and prevalent secondary complication [1]. These ulcers are a leading cause of hospitalization in this population, affecting up to 85-95% of individuals with SCI during their lifetime [6]. This impacts the patient’s quality of life and require careful follow-up and treatment strategies [1].

Available literature remains fragmented across methodological domains including histological, biomechanical, imaging, and clinical assessments. To our knowledge, no previous review has comprehensively mapped these tissue-related changes across methodological approaches and tissue compartments. A scoping review was therefore conducted to synthesize the current evidence base, identify patterns across modalities, and highlight remaining knowledge gaps relevant to clinical management and future research.

## Aim

This article aims to identify, map and describe available evidence on skin and tissue changes following SCI.

## Methods

### Protocol and registration

The review protocol has been published and is available online [7].

### Information sources

The search was executed on March 24, 2025, and included the following databases: MEDLINE (Ovid), EMBASE (Ovid), Cochrane Library, and SCOPUS.

### Search

Table 1 provides a short version of the search strategy, while the complete search strategy for Medline (OVID) database is provided in the supplementary file.

**Table 1:** Short version of search strategy.

Search	Search terms
1	Skin chang* OR skin reaction OR skin symptom OR derma manifestat* OR tissue chang* OR derma* symptom OR derma* chang OR derma* reaction* OR skin manifestation*
2	Skin* OR tissue* OR dermatology OR endothelium OR epidermis* OR sebaceous glands OR sweat glands OR lymph nodes OR membranes OR muscles OR nerve tissue
3	Spinal cord injury OR spinal cord compression OR spinal cord contusion OR spinal cord lesion
4	#1 AND #2 AND #3

### Eligibility criteria

Inclusion criteria were defined as research papers written in English addressing skin changes, dermal changes, tissue changes, and skin manifestations associated with SCI. All study types were eligible for inclusion, while conference abstracts, protocols and opinion pieces were excluded. Reviews were used to examine and verify references.

No restrictions were applied to the publication year, as our aim was to comprehensively incorporate all available literature published in the past. This approach allowed the inclusion of articles exploring fundamental alterations in skin and tissue after SCI, which remain pertinent regardless of publication date.

An academic librarian assisted in refining the search strategy to optimize the balance between sensitivity and precision.

### Selection of sources of evidence

Titles and abstracts were independently screened by two reviewers (LGM, KS). Relevant studies were selected for full-text screening, and any disagreements between the two investigators were resolved through discussion until consensus was reached. Duplicate articles were identified and removed using Covidence, a web-based literature screening and data extraction platform. Full-text articles that met the inclusion criteria were included in the final analysis. Additionally, a backward citation search was performed on the included articles to identify relevant studies not captured in the initial search. EndNote 20 was utilized for reference management, and PRISMA-ScR was used as reporting guideline [8].

### Data extraction

All eligible articles that met the inclusion criteria underwent data extraction by a single reviewer (LGM). The extracted data were subsequently organized into six subgroups based on outcome: 1) histopathological changes, 2) ultrasonographic changes, 3) biomechanical property changes, 4) skin perfusion changes, 5) sebum- and sweat gland excretion changes, and 6) microbiota changes. Subgroups were developed iteratively during data extraction to reflect recurring methodological and biological themes within the literature, and were thus not pre-defined.

Key characteristics of included studies, such as publication year, country of origin, study design, primary outcomes, and sample size, are presented in Table 2.

**Table 2:** Presentation of included studies.

Number	Author	Publication year	Country of origin	Study design	Primary outcome	Study group size
1	Albertin et al.	2018	Italy	Pilot-Cohort	Histopathological changes	3
2	Albertin et al.	2019	Italy	Cohort	Histopathological changes	26
3	Albertin et al.	2022	Italy	Pilot-case	Histopathological changes	16
4	Albuerne et al.	1998	Spain	Cross-sectional	Histopathological changes	30
5	Brunner et al.	2020	Germany	Case-control	Histopathological changes	31
6	Gabison et al.	2019	Canada	Cross-sectional	Ultrasonographical changes	25
7	Hagisawa et al.	1994	USA	Comparative	Skin perfusion changes	20
8	Kuesgen et al.	2009	England	Comparative	Skin perfusion changes	18
9	Li et al.	2010	China	Cross-sectional	Skin perfusion changes	20
10	Makhsous et al.	2007	USA	Cross-sectional	Ultrasonographical changes	30
11	Park et al.	2010	Korea	Case-control	Biomechanical property changes	48
12	Patterson et al.	1993	USA	Comparative	Skin perfusion changes	20
13	Rodriguez et al.	1988	USA	Case-control	Histopathological changes	17
14	Rodriguez et al.	1986	USA	Cross-sectional	Histopathological changes	25
15	Scheel-Sailer et al.	2016	Switzerland	Experimental controlled	Skin perfusion - and biomechanical changes	36
16	Stover et al.	1993	England	Prospective cohort	Histopathological changes	79
17	Stover et al.	1980	England	Case series	Histopathological changes	5
18	Stover et al.	1987	England	Cohort	Histopathological changes	18
19	Stover et al.	1994	England	Prospective cohort	Biomechanical property changes	679
20	Thomas et al.	1984	England	Case-control	Sebum- and sweat gland excretion changes	17
21	Yaggie et al.	2001	USA	Case-control	Sebum- and sweat gland excretion changes	25
22	Yalcin et al.	2013	Turkey	Cross-sectional	Ultrasonographical changes	66
23	Wettstein et al.	2023	Switzerland	Cross-sectional	Microbiota changes	27

### Critical appraisal of individual sources of evidence

In line with scoping review methodology, no formal quality appraisal was performed. The aim of this review was to map the scope and nature of existing evidence rather than to evaluate study rigor.

## Results

**Fig. 1:**
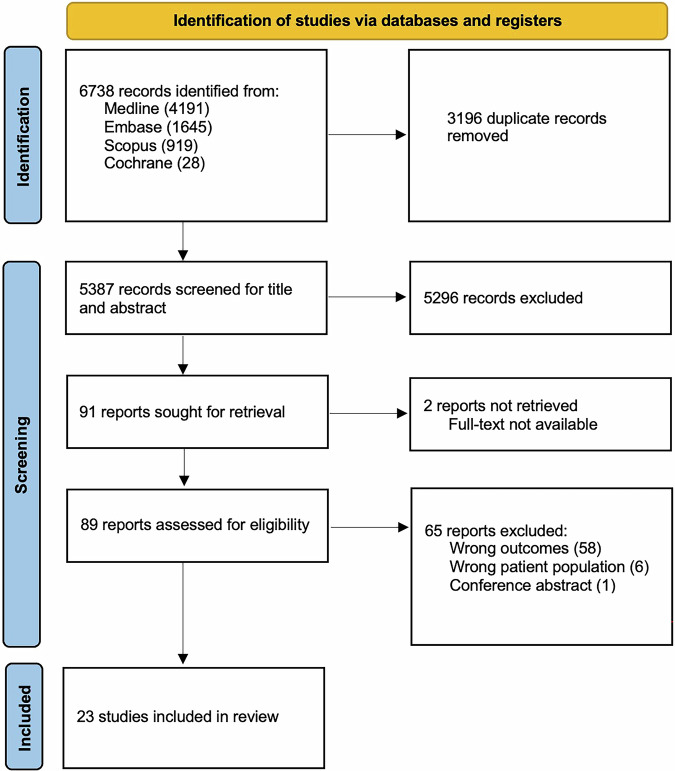
PRISMA flow diagram for inclusion of studies.

A total of 5387 studies underwent title and abstract screening, with 91 studies selected for full-text review. Of these, 23 articles met the inclusion criteria and were included in the final analysis (Fig. 1).

The included articles were categorized in six subgroups based on outcomes as previously described.

### Histopathological changes

Ten articles addressing histopathological changes as the primary outcome were included in this review.

Albertin et al. reported preliminary cohort data based on 6-millimeter skin biopsies from the thighs of three individuals with complete conus medullaris and cauda equina syndromes. The study revealed a significantly thinned and flattened epidermis, measuring 47.6 μm ± 8.8 μm (p = 0.044) [9].

In a subsequent study, Albertin et al. expanded their previous findings to include 26 biopsies and conducted immunohistochemical analyses. They observed a general atrophy, with a thin and flattened epidermis among people with SCI, measuring 46.25 μm ± 1.87 μm. Both epidermal thickness and area progressively decreased over time, starting 1-year post-SCI and continuing until the end of the 8-year follow-up (p = 0.012 and p > 0.0001, respectively) [10].

Additionally, the authors histologically examined the papillae-like structures at the epidermal-dermal junction (interdigitation) by calculating the interdigitation index and the number of papillae-like structures per millimeter in the biopsies. Both parameters showed a significant decline from 1 to 8 years post-SCI (p < 0.0001 for interdigitation index; p = 0.04 for papillae-like structures). Diminished interdigitation and flattening of the dermal-epidermal junction have previously been associated with aging [11, 12]. While no significant differences in Langerhans cell (LC) density were found between groups, a trend towards a reduction in LC density per mm² with increased time post-SCI was noted [10].

In a third study, Albertin et al. focused on differences between individuals with lower motor neuron SCI (LMNSCI) and upper motor neuron SCI (UMNSCI). LMNSCI individuals exhibited progressive epidermal thinning over time and a significant reduction in LC density, suggesting long-term skin immunosuppression lasting up to 10 years. In contrast, UMNSCI individuals retained nearly twice the LC density of LMNSCI individuals, indicating less immune compromise. This highlights the greater structural and immune deficits caused by permanent denervation in LMNSCI [13].

Albuerne et al. conducted a cross-sectional study investigating the presence and modification of S100-alpha and S100-beta proteins in sensory corpuscles of people with SCI. S100 proteins regulate intracellular calcium homeostasis and act as trigger/activator proteins in neurons. Their study found that S100 immunoreactivity in sensory corpuscles remains unchanged in individuals with SCI compared to healthy controls [14].

Brunner et al. conducted a case-control study to examine the cellular and molecular changes in intact skin affected by SCI [15].

Through immunostaining, they discovered that platelets were present outside of blood vessels, scattered throughout the dermis, and clustered in small groups in the intact skin of individuals with SCI. This indicates peripheral leakage of plasma components, such as platelets, into the dermis. This phenomenon is characteristic of the early wound healing process and is also observed in chronic SCI skin [15].

Additionally, transforming growth factor (TGF)-beta1 protein levels were elevated in the dermis of SCI-affected skin compared to controls, with accumulation observed at the sub-epidermal junction zone. Since growth factors like TGF-beta1 are released by activated platelets, this suggests a series of linked events. The in vivo activity of TGF-beta was significantly higher in SCI-affected skin compared to controls, as assessed by the plasminogen activator inhibitor-1 (PAI-1) bioassay. Moreover, Inhibin Subunit Beta A (INHBA) mRNA expression, a marker for Activin, was significantly increased [15]

Rodriguez et al. investigated differences in amino acid composition and the activity of the collagen biosynthesis enzyme lysyl hydroxylase in affected and unaffected skin from individuals with SCI. They found that the amino acid content in insensitive skin was significantly decreased, and the hydroxylation rate of proline (the proline/hydroxyproline ratio) was lower, along with a reduction in lysyl hydroxylase activity. These changes suggest a lower resistance to mechanical stress, as collagen is critical for skin tensile strength [16].

Stover et al. describe a case report involving four individuals with lesions above the T6 level, noting the presence of brawny induration of the skin. They highlight that injuries at the T4-T6 level are critical because they result in the loss of sympathetic nervous system responses and subsequent autonomic hyperreflexia. Skin biopsies from these individuals with lesions above T6 revealed atrophic epidermis and a notable decrease in Type III collagen in the upper dermis, while Type I collagen distribution remained unchanged [17].

Type III collagen is known for its elastic properties and role in forming elastic networks, whereas Type I collagen is a stiff fibrillar protein that provides tensile strength [18]. Additionally, no cholinesterase activity was observed in the dermis around hair follicles or touch receptors, though sparse activity was found around blood vessels, indicating a lack of functional cholinergic innervation [17].

In a cohort study, Stover et al. finds that seventeen out of eighteen individuals presenting dermal fibrosis by clinical observation showed a histological reduction in Type III collagen in the upper dermis with an almost exclusively appearance of type I fibers in the entire dermis, with a “clumped” appearance. They describe a similar appearance of collagen distribution in the fibrotic stage of progressive systematic sclerosis. All individuals had injury above T6 segment. Interestingly, two individuals with lesions below T6 showed no dermal fibrosis and none of the above histological features, indicating a strong correlation of dermal fibrosis to autonomic dysfunction, as this is well known to be a predisposor [19].

Stover et al. also performed a prospective cohort study examining skin biopsies from individuals with both chronic and acute SCI. They identified dermal fibrosis and perivascular infiltration in their findings. In the chronic group, dermal fibrosis was observed more frequently in individuals with tetraplegia (65%) compared to those with paraplegia (25%), and it was more common when clinical skin thickening was present. In the acute group, dermal fibrosis was found in 14 out of 20 samples from individuals only two months post-SCI. Scanning electron microscopy revealed no significant differences in collagen arrangement or size when compared to controls; however, specific analysis and determination of collagen types I-III were not performed [20].

Rodriguez et al. investigated differences in alpha- and beta-adrenergic receptors in 25 skin biopsies from individuals with SCI. The individuals were divided into “early” and “late” groups, based on whether they were under or over five years post-injury. While no statistically significant differences were found due to the small sample size and large variation, the authors noted a clear trend towards a reduction in adrenergic receptors with increasing time since injury [21].

### Ultrasonographical changes

Three cross-sectional studies evaluating skin and tissue changes using ultrasound were included.

Gabison et al. used ultrasound imaging to study the skin and subcutaneous tissue at the tuber ischiadicum. Their findings revealed no significant differences in the thickness of the skin or subcutaneous tissue between the groups studied [22].

Maksouhs et al. utilized the Tissue Ultrasound Palpation System (TUPS) to assess tissue stiffness and thickness in various anatomical sites of individuals with SCI and able-bodied individuals. While no significant differences in soft tissue thickness were observed at selected high-risk pressure ulcer sites between the groups, tissue stiffness were significantly lower in the SCI group at the ischial tuberosity (IT) and posterior mid-thigh (MT) (IT: p = 0.035; MT: p = 0.001) [23].

Yalcin et al. used ultrasound to measure skin thickness differences between individuals with SCI and controls. They found significantly thinner skin in the SCI group at the sacrum (2.1 mm ± 0.9 mm vs. 3.2 mm ± 0.5 mm, p < 0.05) and the ischial tuberosity (2.2 mm ± 0.6 mm vs. 2.6 mm ± 0.5 mm, p < 0.05) [24].

### Biomechanical property changes

Three articles exploring changes in the biomechanical skin and tissue properties after SCI were included in this review.

In a case-control study, Park et al. used a Cutometer, a non-invasive suction device, to assess biomechanical skin properties, including distensibility, elasticity, and viscoelasticity. They found altered biomechanical properties in the skin of individuals with SCI. Subgroups with sympathetic paralysis, regardless of somatic sensory completeness, exhibited lower skin distensibility and higher values of elasticity and viscoelasticity. The study concludes that sympathetic paralysis has a more significant impact on these skin properties than somatic sensory paralysis [25].

Scheel-Sailer conducted an experimentally controlled study comparing the biophysical skin properties of individuals with SCI to those of controls. The study found no significant differences in skin elasticity between the two groups [26].

Stover et al. performed a prospective study involving 679 individuals with SCI, where skin thickening was measured using the pinch test by two reviewers. They found that skin thickening occurred in 57.9% of individuals with cervical injuries, 31.6% with upper thoracic injuries, 23.6% with lower thoracic injuries, and 16.0% with thoracolumbar injuries (p < .0001). The prevalence of skin thickening increased with time post-injury, affecting 20.3% of individuals at 1 year and 51.2% at 5 years, with a plateau observed after 5 years (p < .0001). Additionally, skin thickening was correlated with autonomic dysreflexia [27]. Clinically observed skin thickening likely reflects dermal fibrosis, altered collagen organization, and reduced tissue compliance rather than epidermal hypertrophy. This interpretation aligns with histological findings demonstrating epidermal thinning alongside collagen remodeling and fibrotic changes within deeper dermal layers.

### Skin perfusion changes

This review included five articles examining skin perfusion changes following SCI.

In a comparative study, Hagisawi et al. investigated reactive hyperemia responses after repeated pressure loads in individuals with SCI and controls. They found no significant differences in reactive hyperemia responses between the two groups [28].

Kuesgen et al. conducted a comparative study using laser Doppler fluxmetry to evaluate sensory axon-reflex vasodilatation in individuals with SCI. They found a 39% reduction in axon-reflex vasodilatation in individuals with SCI compared to controls, although the response remained detectable in all cases [29].

In a cross-sectional study, Li et al. investigated oscillations in tissue oxygenation at the sacral region in individuals with SCI and controls during rest, during pressure loading, and post-loading using wavelet analysis at different frequencies. They found significant differences in the spontaneous skin tissue [HbO2] and [Hb] signals during both the rest and preload periods. This suggests a reduced ability for metabolic regulation of blood flow in the skin to meet cellular needs in individuals with SCI, resulting in decreased oxygen delivery.

Additionally, neurogenic activity in the skin of individuals with SCI was found to be lower during pre- and post-loading periods, indicating a reduced neurogenic contribution to skin oxygenation regulation. Consequently, the reactive hyperemia typically observed following pressure loads was diminished in individuals with SCI, leaving the skin without adequate protective mechanisms against pressure loads, leading to insufficient tissue recovery [30].

Patterson et al. compared the effects of pressure loads on skin perfusion between able-bodied individuals and individuals with SCI. They observed a 77% decrease in transcutaneous skin PO2 in individuals with SCI after a 30 mmHg pressure load, compared to a 14% decrease in the able-bodied group, indicating impaired autoregulation and reduced skin perfusion in individuals with SCI. Laser Doppler measurements further supported these findings, showing reduced blood flow volume in the SCI group after pressure loading [31].

Scheel-Sailer et al. conducted a biophysiological study to assess skin hydration, redness, elasticity, and perfusion. While no significant differences were observed in skin hydration or elasticity between individuals with SCI and controls, skin perfusion was unexpectedly 74% higher in SCI skin compared to controls. The authors suggest that this discrepancy might be due to increased skin irritation or heightened susceptibility to shear and pressure loads during individuals examination. Additionally, altered skin redness in the sacral region were noted in individuals with SCI compared to controls, potentially indicating early signs of skin irritation from pressure loads [26].

### Sebum- and sweat gland excretion changes

The review included two articles exploring changes in sebum and sweat gland excretion.

Thomas et al. demonstrated in their case-control study that individuals with paraplegia had a sebum excretion rate twice as high compared to controls at affected sites (p < 0.001). No significant difference in sebum excretion was observed between groups at the forehead location. The authors suggest that this increased sebum production might contribute to the higher incidence of acne vulgaris in individuals with SCI at affected sites [32].

In a case-control study, Yaggie et al. investigated sweat excretion rates in individuals with SCI with injuries from C4 to C8. They found decreased sweat production in individuals with tetraplegia compared to controls, which they attributed to sympathetic decentralization [33].

### Microbiota changes

The study included a single article examining changes in skin microbiota.

In a cross-sectional study, Wettstein et al. examined the skin microbiome below the lesion level in individuals with SCI and compared it to above the lesion level and to able-bodied individuals. They found no difference in the median number of bacterial genera between the pelvic microbiome of individuals with SCI and the shoulder microbiome of controls. However, significant differences in skin microbiome composition were observed between the pelvic and shoulder locations, with Staphylococcus identified as the dominant genus at both sites. The level of SCI lesion did not influence the skin microbiome composition [34].

## Discussion

**Fig. 2:**
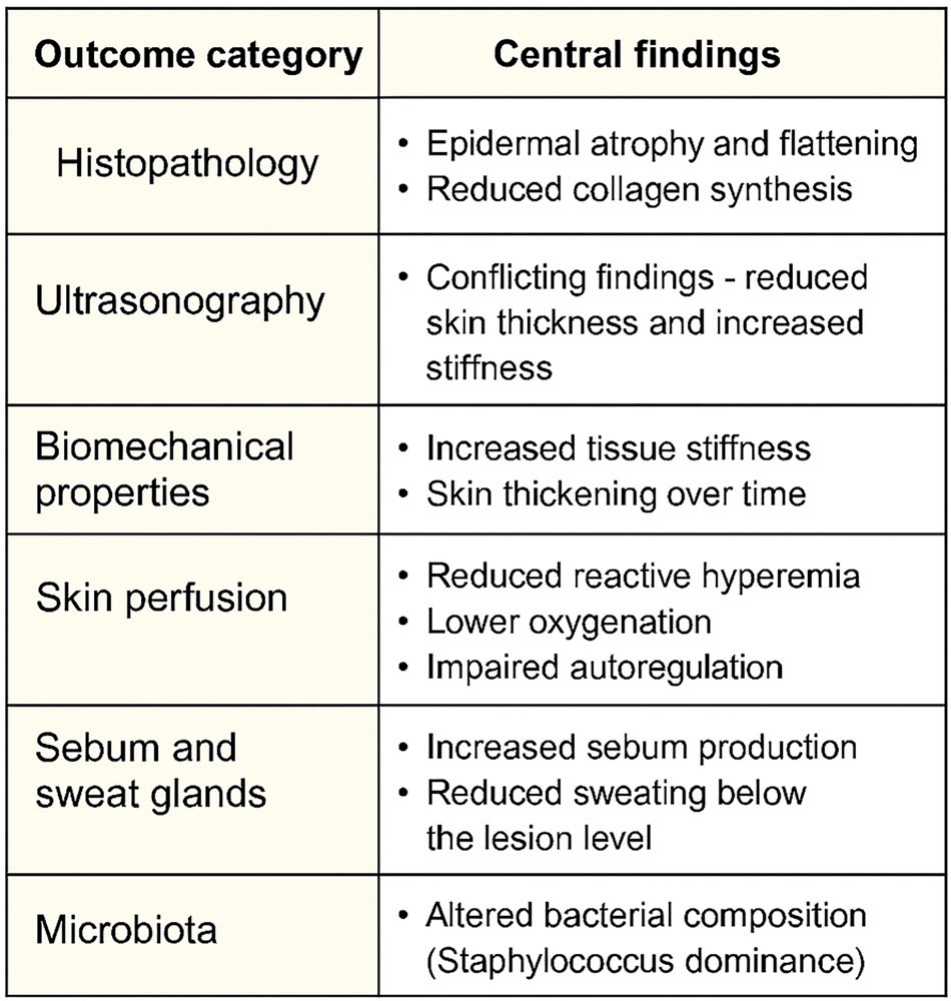
Central findings based on outcome category. Stiffness-related findings varied by assessment modality and tissue depth, with reduced bulk soft-tissue stiffness but increased superficial dermal fibrosis and reduced compliance.

The main findings suggest that skin affected by SCI undergoes progressive thinning and atrophy, reduced collagen synthesis, impaired vascular response, and alterations in biomechanical properties. Collectively, these changes compromise the skin’s structural integrity and physiological function, thereby increasing vulnerability to pressure ulcers and delaying wound healing (Fig. 2). The discussion below is organized according to the primary result categories.

### Histopathological changes

Studies included in our review consistently report epidermal thinning and flattening in individuals with SCI, suggesting that SCI accelerates skin degeneration (8, 9, 12). The reduction in epidermal thickness and the decrease in interdigitation at the dermal-epidermal junction compromise the skin’s mechanical stability and regenerative capacity, increasing the risk of pressure ulcers and delayed wound healing. individuals with LMNSCI are particularly affected, exhibiting more severe epidermal atrophy and immune deficits than individuals with UMNSCI, likely due to permanent denervation.

The observed reduction in LC density in individuals with LMNSCI suggests a prolonged impairment of the skin’s immune function, persisting for up to a decade post-injury [13]. This immunosuppression increases vulnerability to infections and impairs wound healing. Conversely, individuals with UMNSCI demonstrate relatively preserved LC density, indicating less compromised immune function. This discrepancy highlights the differential impact of SCI types on skin immunity and suggests that individuals with LMNSCI may require more intensive monitoring and prophylactic measures to prevent infections.

Changes in collagen composition further characterize SCI-affected skin. Studies by Stover et al. and Rodriguez et al. revealed a significant reduction in Type III collagen, critical for skin elasticity, and an increased presence of Type I collagen, known for its tensile strength [16, 17, 19, 20]. This shift contributes to dermal fibrosis and reduced skin flexibility, mirroring fibrotic patterns seen in systemic sclerosis. The association between autonomic dysfunction and collagen remodeling, particularly in individuals with lesions above T6, highlights that sympathetic nervous system disruption plays a role in these fibrotic changes. Elevated TGF-beta1 levels, as reported by Brunner et al., further indicate ongoing inflammatory and fibrotic processes in SCI-affected skin [15].

The histopathological changes observed in SCI-affected skin are similar to those seen in aging skin, including epidermal thinning and reduced dermal-epidermal interdigitation. Progressive epidermal atrophy and a decline in LC density weaken the skin’s protective and immunological functions, increasing vulnerability to mechanical stress, infections, and delayed wound healing. Further longitudinal studies are required to elucidate the temporal progression of histopathological changes and to explore interventions that could mitigate these effects.

### Ultrasonographic changes

Ultrasound studies revealed inconsistent findings regarding skin thickness in individuals with SCI. While some studies reported significant thinning, particularly in areas prone to pressure ulcers, such as the sacrum and ischial tuberosities, others found no significant differences compared to controls. Similarly, results on subcutaneous tissue thickness varied across studies, likely reflecting methodological variability or differences in patient populations. Tissue stiffness, however, was consistently reported to be lower in SCI-affected areas. Reduced tissue stiffness compromises the skin’s ability to withstand pressure and shear forces. These findings emphasize the importance of developing standardized ultrasound protocols and conducting larger, more homogeneous studies to confirm these observations and clarify their clinical implications.

### Biomechanical property changes

Alterations in the biomechanical properties of SCI-affected skin, including reduced distensibility and increased stiffness, were more pronounced in individuals with sympathetic paralysis. These findings suggests that autonomic dysfunction plays a role in skin remodeling post-SCI. Such changes compromise the skin’s ability to distribute mechanical forces, increasing the risk of pressure ulcers and delayed wound healing [35]. Interestingly, while some studies reported no significant differences in skin elasticity between individuals with SCI and controls, others reported substantial changes, particularly in areas exposed to prolonged pressure. These inconsistencies may arise from variations in injury levels, time since injury, and assessment techniques, highlighting the need for more standardized methodologies. The lack of longitudinal studies further limits our understanding of how biomechanical properties evolve over time post-injury. Future research should aim to clarify these discrepancies by incorporating larger sample sizes, consistent assessment tools, and follow-up periods to better inform clinical management and preventive strategies.

### Skin perfusion changes

Reduced skin perfusion and impaired vascular responses were consistent findings across the reviewed studies. Diminished reactive hyperemia, reduced neurogenic regulation, and impaired autoregulation contribute to inadequate oxygen and nutrient delivery to the skin, compromising its ability to recover from mechanical stress. These perfusion deficits are critical factors in the pathogenesis of pressure ulcers, as they leave the skin vulnerable to ischemia and delayed wound healing. Notably, one study reported higher skin perfusion, which the authors attributed to reactive changes or potential skin irritation during measurement. Such findings may also reflect methodological differences or variations in injury characteristics, underscoring the need for standardized protocols in the future. Understanding the mechanisms underlying these vascular changes, including autonomic dysregulation and microvascular impairments, could inform therapeutic strategies. Further research is needed to establish guidelines for optimizing vascular health in individuals with SCI.

### Sebum and sweat gland changes

The increase in sebum excretion and the decrease in sweat gland function observed in individuals with SCI reflect the disrupted autonomic control in SCI-affected skin. Elevated sebum production could explain the higher prevalence of acne in SCI individuals, whereas reduced sweating contributes to thermoregulation issues and skin dryness. These findings underscore the importance of managing skin hydration and sebum regulation in clinical care plans for individuals with SCI. Addressing these changes may prevent secondary complications such as infections or pressure ulcers [1, 35, 36].

### Microbiota changes

The limited evidence on skin microbiota indicates no significant differences in bacterial diversity but notable differences in composition across different body regions. This suggests that factors such as skin moisture, perfusion, and barrier function may influence microbiota distribution post-SCI. Further research is needed to understand how these changes impact wound healing and infection risk, potentially guiding probiotic or antimicrobial treatments.

## Spinal cord injury and ageing

Several of the structural and biomechanical alterations identified in this review resemble well-described features of skin ageing. Comparing SCI-affected skin with ageing skin may therefore provide a useful biological framework for interpreting the observed tissue changes and their potential clinical consequences. Aging skin thins progressively, losing approximately 6.4% of epidermal thickness per decade due to reduced epidermal cell numbers and overall atrophy [37]. Similarly, SCI-affected skin exhibits thinning and atrophy, compromising structural integrity in both cases.

Aging also flattens the dermo-epidermal junction, reducing resistance to shearing forces and increasing vulnerability to damage [38, 39]. individuals with SCI experience similar flattening, contributing to higher pressure ulcer risk. Additionally, aging leads to reduced dermal thickness, collagen turnover, and elastin integrity, causing rigidity, decreased elasticity, and slower recovery from mechanical stress [38, 39]. SCI-affected skin mirrors these changes with increased stiffness and diminished collagen synthesis, underlining the shared impact of collagen and elastin degradation.

## Clinical implications

The cumulative evidence underscores a heightened risk of skin-related complications in individuals with SCI, particularly those with LMNSCI or lesions above T6. Progressive epidermal atrophy, immune suppression, and connective tissue remodeling contribute to an increased likelihood of pressure ulcers, chronic wounds, and infections. Clinicians should adopt a proactive, individualized approach to skin care in individuals with SCI, focusing on early detection and management of skin changes, especially in high-risk populations. Preventing pressure ulcers is crucial and requires early risk assessment, frequent repositioning, routine skin checks, and the use of advanced support surfaces. Targeted skin care, including moisturizing to address dryness and managing sebum production, can help maintain skin integrity and prevent infections.

Educating patients and caregivers about skin inspection and pressure relief techniques empowers proactive care and reduces the risk of complications. Patients and healthcare professionals should also have knowledge about the significantly high risk of pressure ulcers due to skin changes following SCI. Additionally, the use of leveraging technology, such as wearable sensors and telehealth, could support real-time monitoring, improve access to specialized treatment and care, and enhance overall patient outcomes.

These findings underscore the need for tailored clinical management to address the progressive skin changes observed in individuals with SCI. By understanding the mechanisms of epidermal atrophy, immune dysfunction and collagen remodeling, clinicians can develop targeted therapies to mitigate skin complications post-SCI and improve patient care.

## Limitations

This review aimed to provide a comprehensive overview of skin and tissue changes following SCI. While the included studies provide valuable insights, several limitations must be acknowledged. The included studies demonstrated substantial heterogeneity in sample size, injury level, follow-up duration, and methodological approaches, which complicates synthesis and limits the generalizability of findings. Variations in SCI type (e.g., upper versus lower motor neuron injuries) and patient demographics further contribute to the complexity. Another limitation is that despite a systematic approach, the review cannot guarantee identification of all relevant studies. Future research should prioritize longitudinal studies with larger well-characterized cohorts to elucidate the progression of these changes over time. Additionally, exploring underlying molecular mechanisms, such as the involvement of adrenergic receptors and growth factors, may support the development of targeted therapeutic strategies aimed at enhancing skin health and resilience in individuals with SCI.

## Conclusion

This scoping review identifies significant structural, functional, and biochemical changes in the skin following SCI, many of which are parallel to those observed in aging. These findings emphasize the critical need for targeted interventions to mitigate the increased vulnerability in SCI-affected skin to complications such as pressure ulcers and infections. Future research should focus on high-quality, longitudinal studies to better understand the mechanisms driving these changes and to develop evidence-based strategies for prevention and treatment of skin-related complications. By addressing these gaps, researchers and clinicians can work toward improving the quality of life and clinical outcomes for individuals living with SCI.

## Supplementary information

Supplemental Material

## Data Availability

Data sharing not applicable to this article as no datasets were generated or analysed during the current study.
